# De-escalation yes, but not at the expense of efficacy: in defense of better treatment

**DOI:** 10.1038/s41523-019-0120-z

**Published:** 2019-08-12

**Authors:** Charles L. Shapiro

**Affiliations:** Department of Medicine, Director of Translational Breast Cancer Research, Director of Cancer Survivorship, Icahn School of Medicine, Mount Sinai, NY 10029 USA

**Keywords:** Breast cancer, Medical research

Most chemotherapy regimens for breast cancers are empiric. Based on historical precedents or incremental additions, the specific doses and durations are such that the side-effects are tolerable, and the benefits are tangible. More than 50 years of randomized trials in tens of thousands of women establish that adjuvant chemotherapy unequivocally decreases breast cancer mortality.^1–3^ These adjuvant chemotherapy regimens have stood the test of time, and many are in use today. The best example of chemotherapy de-escalation is no chemotherapy, which has been prospectively validated using the Oncotype DX recurrence score in the Tailor X trial^4,5^ and the 70-gene panel in the MINDACT trial.^6^

The first polychemotherapy regimen was “classical” CMF (cyclophosphamide (100 mg/M2 oral days 1–4), methotrexate (intravenous (IV) 40 mg/M2 days 1 and 8), and fluorouracil (IV 600 mg/M2 days 1 and 8) on a 28-day schedule for 12 treatment cycles.^7,8^ Compared to no treatment control, adjuvant CMF statistically significantly improved disease-free (DFS) and overall survival (OS). De-escalation to six treatment cycles of CMF was the result of a randomized trial of comparing six versus 12 cycles.^9^ Classical CMF was modified to all IV regimen in the adjuvant setting, however, in the metastatic disease setting classical CMF is superior to IV CMF, likely because of increased dose intensity of the classic regimen.^10^

The incorporation of anthracyclines was the next incremental step in adjuvant chemotherapy.^11^ From randomized trials,^12,13^ and the results of a meta-analysis performed by the Early Breast Cancer Collaborative Trialist Group (ECBTG),^3^ four cycles of standard AC (doxorubicin 60 mg/M2 and cyclophosphamide 600 mg/M2) every 21 days are comparable to classical CMF at 10 years. What differs between these regimens is the duration of chemotherapy (3 and 6 months for AC and CMF, respectively) and the side-effects.^14^

The next incremental step was the introduction of the taxanes.^2^ In a randomized trial of over 1000 women, TC (docetaxel 75 mg/M2 and cyclophosphamide 600 mg/M2) given every 21 days for four cycles is statistically significantly superior to AC in DFS and OS at 7 years of median follow-up.^15^ The trial population was of a median age 52, 69% were hormone-receptor positive, and 48% were node-negative. The magnitude of the absolute benefits was 6% in DFS (hazard rate 0.74 (95% CI 0.56–0.98), *p* = 0.033) and 5% in overall survival (HR = 0.69 (95% CI 0.50–0.97, *p* = 0.032).^15^ Also, the benefits were independent of age and hormone receptor status. Other analyses show that TC is cost-effective relative to AC.^16,17^

No randomized trial directly compares TC to CMF. Based on indirect comparisons, which are subject to uncertainty and bias,^18^ TC is superior to CMF. Despite the inherent uncertainty, policy-making and drug reimbursement organizations accept adjusted indirect comparisons.^18^ While it is a leap of faith to consider TC superior to CMF, others have made this leap. A network meta-analysis showed that TC was statistically significantly superior to CMF and AC in event-free and overall survival, and was most comparable to AC plus paclitaxel.^19^ However, TC has more side-effects than CMF, particularly hematologic events. Finally, in the National Comprehensive Cancer Network Breast Cancer Guidelines TC is a “preferred” regimen in the same category as AC followed by paclitaxel, whereas CMF is “useful in certain situations.” (www.nccn.org/professionals/physician_gls/pdf/breast.pdf).

The major side-effects of TC (grades 3 and 4) were anemia-2%, thrombocytopenia-1%, neutropenia-51%, febrile neutropenia-5%, asthenia-4%, edema-1%, fever-5%, infection-8%, myalgia-2%, nausea-16% (include grades 2–4), vomiting-7% (includes grades 2–4), stomatitis-2%, phlebitis-1%, and alopecia in more than 90%.^20^ Whereas side-effects with CMF (grades 3 and 4) were neutropenia-9%, thrombocytopenia-1%, infection-2%, nausea-43% (includes grades 2–4), vomiting-43% (includes grades 2–4) diarrhea-5%, phlebitis<1%, and alopecia-40%.^14^ The high incidence of neutropenia (51%) associated with TC was without peg-filgrastim, which is now routine in women receiving TC.^21^ Likewise, the high incidence of nausea and vomiting with CMF was most likely before newer antiemetic drugs.^22^ Quality-of-life decreases during and right after completing adjuvant chemotherapy but then improves, although certain side-effects may persist.^23^ Studies of long-term breast cancer survivors show that quality-of-life is comparable between those who did and did not receive chemotherapy.^24,25^ Thus, the acute toxicities mostly resolve after adjuvant chemotherapy and in the long-term quality-of-life gets better.

It is illuminating when patients are asked to rate their toxicities,^26,27^ or express their preferences about the benefits of adjuvant chemotherapy.^28^ Seventy-seven percent of women would consider it worthwhile to receive 6-months of adjuvant CMF for a 1-year increase in survival (about 3% increase in survival).^28^ Another study shows that over 50% of women with breast cancer treated with CMF would take adjuvant chemotherapy for one day or 0.1% survival increase.^29^ Thus, women have a low threshold of benefit when they are asked about adjuvant chemotherapy.

The benefits of adjuvant chemotherapy are comparable irrespective of nodal status.^2^ What differs is the underlying risk of distant recurrence. Based on genomic assays we know which women will benefit from adjuvant chemotherapy. Pre-genomic testing, the principal argument for choosing CMF over TC in “low risk” node-negative women is TC exposes all the population to more side-effects when only relatively few will benefit. In the era of genomic testing, a plausible rationale for choosing TC is that it is more effective therapy despite some increased toxicity, and the treatment duration is only three months as opposed to six months.

Shared decision making is an aspirational goal,^30,31^ although it is one of the cornerstones of patient-centered care. TC versus CMF represents an opportunity for shared decision making. Imagine two women: one with Oncotype recurrence score of 25 (10-year distant recurrence rate of 16%) and one with a recurrence score of 50 (10-year distant recurrence of 33%). There are two assumptions in this theoretical analysis. First, distant-disease is a surrogate for death, which is true most instances especially in young women and a significant proportion of older women without competing co-morbidities.^32^ The HR for OS is 0.69 for TC versus AC.^15^ Since AC is comparable CMF, the second assumption is also that the HR is also 0.69 for TC versus CMF.

Table 1 describes the analysis. The mortality reductions are double in TC relative to CMF. It is reasonable to engage in shared decision-making in the case of the woman with a 16% 10-year distant recurrence rate. The shorter regimen (TC) with more benefits but some increased side-effects versus the more extended regimen (CMF) with fewer benefits and fewer side-effects. In the case of the woman 10-year distant recurrence of 33%, TC is undoubtedly the better option. It is important to remember that the acute toxicities of adjuvant chemotherapy mostly resolve, quality-of-life improves, and women will go through adjuvant chemotherapy for little gain in survival.

**Table 1:** Absolute mortality reductions of TC relative to CMF

Recurrence score	10-year of distant metastases	1-HR × recurrence score	Distant recurrence (deaths)/100 women
25	16%	0.31 × 16 = 5	5/100
50	33%	0.31 × 33 = 10	10/100

*TC* docetaxel and cyclophosphamide, *CMF* cyclophosphamide, methotrexate, and fluorouracil, *HR* hazard ratio

Incomplete data fuel the conundrum of TC versus CMF. There is no randomized trial directly comparing the two regimens nor is there one likely to be in the future. Purists may consider the uncertainty of indirect comparisons of TC and CMF, and increased side-effects of TC despite its shorter duration, making CMF the preferred option. Whereas the practicalists accept the indirect comparisons, because that is the only available data, and recommend TC because of its superior efficacy and shorter duration. Individual women may have equally strong preferences for one regimen over the other, and health care professionals should listen and trust their patients to make the right decision while providing guidance and making a recommendation.

## Data Availability

The data presented in Table 1 are simulated.
